# Mitochondrial abnormalities and disruption of the neuromuscular junction precede the clinical phenotype and motor neuron loss in hFUS^WT^ transgenic mice

**DOI:** 10.1093/hmg/ddx415

**Published:** 2017-11-28

**Authors:** Eva So, Jacqueline C Mitchell, Caroline Memmi, George Chennell, Gema Vizcay-Barrena, Leanne Allison, Christopher E Shaw, Caroline Vance

**Affiliations:** 1 Department of Basic and Clinical Neuroscience; 2 Wohl Cellular Imaging Centre, Institute of Psychiatry, Psychology and Neuroscience, King’s College London, Denmark Hill, London SE5 8AF, UK; 3 Centre for Ultrastructural Imaging, King’s College London, New Hunts House, Guy’s Campus, London SE1 1UL, UK

## Abstract

FUS (fused in sarcoma) mislocalization and cytoplasmic aggregation are hallmark pathologies in FUS-related amyotrophic lateral sclerosis and frontotemporal dementia. Many of the mechanistic hypotheses have focused on a loss of nuclear function in the FUS-opathies, implicating dysregulated RNA transcription and splicing in driving neurodegeneration. Recent studies describe an additional somato-dendritic localization for FUS in the cerebral cortex implying a regulatory role in mRNA transport and local translation at the synapse. Here, we report that FUS is also abundant at the pre-synaptic terminal of the neuromuscular junction (NMJ), suggesting an important function for this protein at peripheral synapses. We have previously reported dose and age-dependent motor neuron degeneration in transgenic mice overexpressing human wild-type FUS, resulting in a motor phenotype detected by ∼28 days and death by ∼100 days. Now, we report the earliest structural events using electron microscopy and quantitative immunohistochemistry. Mitochondrial abnormalities in the pre-synaptic motor nerve terminals are detected at postnatal day 6, which are more pronounced at P15 and accompanied by a loss of synaptic vesicles and synaptophysin protein coupled with NMJs of a smaller size at a time when there is no detectable motor neuron loss. These changes occur in the presence of abundant FUS and support a peripheral toxic gain of function. This appearance is typical of a ‘dying-back’ axonopathy, with the earliest manifestation being mitochondrial disruption. These findings support our hypothesis that FUS has an important function at the NMJ, and challenge the ‘loss of nuclear function’ hypothesis for disease pathogenesis in the FUS-opathies.

## Introduction

Amyotrophic lateral sclerosis (ALS) and frontotemporal dementia (FTD) are fatal neurodegenerative diseases that share many clinical, pathological and genetic features. Mutations in many genes are implicated in the pathogenesis of both diseases, including the gene encoding *Fused in Sarcoma* (FUS), which accounts for ∼5% of familial ALS cases (1–3). FUS mutations occur predominantly in the C-terminal and disrupt the nuclear localising signal (NLS) leading to cytoplasmic mislocalization and aggregation of FUS protein (4,5). Cytoplasmic inclusions of wild-type FUS protein also occur in ∼5% of FTD cases, when it co-localises with the functionally similar proteins Ewing’s Sarcoma (EWS) and TAF15 (6). This is in contrast to ALS-FUS where mutant FUS aggregates occur in the absence of these proteins (7). We have previously shown that transgenic mice overexpressing human wild-type FUS develop cytoplasmic aggregates of FUS in the brain and spinal cord, leading to progressive hind limb paralysis and motor neuron degeneration in an age and dose-dependent manner (8).

FUS is a predominantly nuclear protein involved in many aspects of RNA processing (9). However, a growing body of evidence now points towards a cytoplasmic role of FUS in the transport, stability and local translation of mRNA at the synapses. Studies in hippocampal neurons revealed that FUS associates with mRNA encoding an actin-stabilising protein Nd1-L and facilitates its transport to dendritic spines (10–12). A study in transgenic mice demonstrated that mutant FUS induces motor neuron degeneration preceded by early abnormalities at the neuromuscular junction (NMJ) through a toxic gain of function (13). Another recent study reported that FUS knockdown reduced synaptic transmission both in cultures and *in vivo*, resulting in behavioural aberrations in FUS knockdown mice (14). These studies appear to link impaired cytoplasmic function of FUS to synaptic dysfunction and neurodegeneration.

In many neurodegenerative diseases, synaptic dysfunction and disruption are some of the earliest pathological events that occur long before neuronal loss. Accumulating evidence suggests that degeneration of the NMJ is an early feature of ALS and may contribute to motor neuron death (15–17). Recent studies in animal models and ALS patients suggest a progressive distal to proximal ‘dying-back’ disease mechanism, implicating neuromuscular synaptic vulnerability prior to clinical manifestation (15,16,18,19). The vast majority of animal model studies explore the effects of mutant SOD1 models, and relatively little is known about disease mechanisms in mouse models of other ALS-linked genes such as TDP-43 and FUS.

Here, we report that FUS is abundant at the pre-synaptic terminal at the NMJ in non-transgenic mice implying that it plays an important role in synaptic function. Mice over-expressing human wild-type FUS develop significant loss of NMJs by post-natal day 15, which precedes motor neuron loss and is associated with dramatic ultrastructural changes including mitochondrial vacuolation and a loss of synaptic vesicles. These results indicate that FUS plays an important role at the NMJ, and that overexpression of FUS leads to a ‘dying-back’ axonal and subsequent motor neuron degeneration.

## Results

### FUS is abundant at the neuromuscular junctions

**Figure 1.**
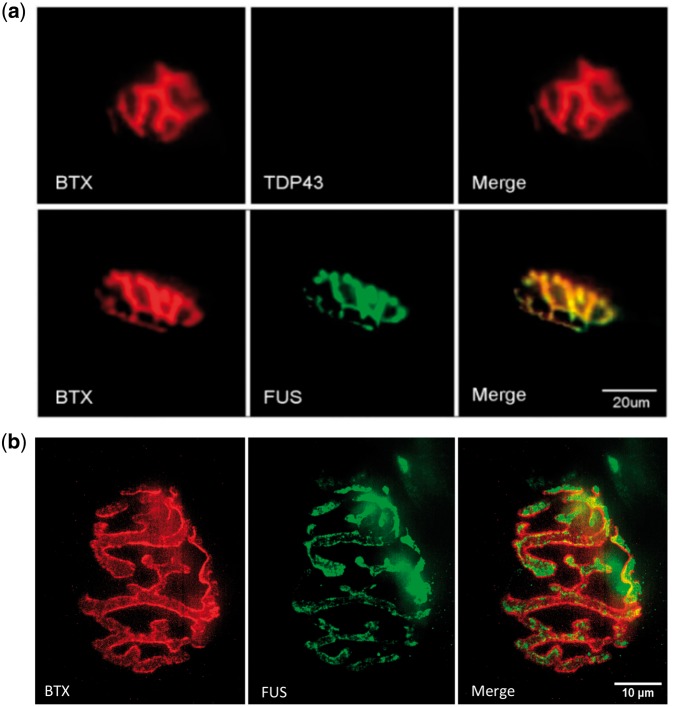
FUS is abundant at the neuromuscular junction in non-transgenic mice. (**A**) FUS (green) is abundant and co-localised with BTX (red) whereas TDP-43 is absent at the NMJ in adult NTg mice (scale bar: 20 μm). (**B**) Super resolution microscopy of the NMJ shows diffuse FUS (green) surrounded by BTX (red) demonstrating clear separation between the pre-synaptic (FUS) and post-synaptic (BTX) sides of the NMJ (scale bar: 10 μm).

Immunohistochemistry revealed a perfect co-localization of FUS with the NMJ marker α-bungarotoxin (BTX) in the gastrocnemius muscle in NTg adult mice (Fig. 1A). Similar investigation of another ALS linked RNA binding protein, TDP-43, showed no such localization to the NMJ (Fig. 1A) in NTg mice, suggesting that FUS has a specific role at the NMJ that is distinct from TDP-43. In order to identify whether FUS was localised to the pre- or post-synaptic side of the NMJ, we performed super resolution microscopy to compare the FUS and BTX staining (Fig. 1B). We observed diffuse cytoplasmic FUS staining within the nerve terminal surrounded by but not overlapping the BTX stain. Given that the BTX stains post-synaptic acetylcholine receptors, this confirms that FUS is localised to the pre-synaptic side of the NMJ.

**Figure 2.**
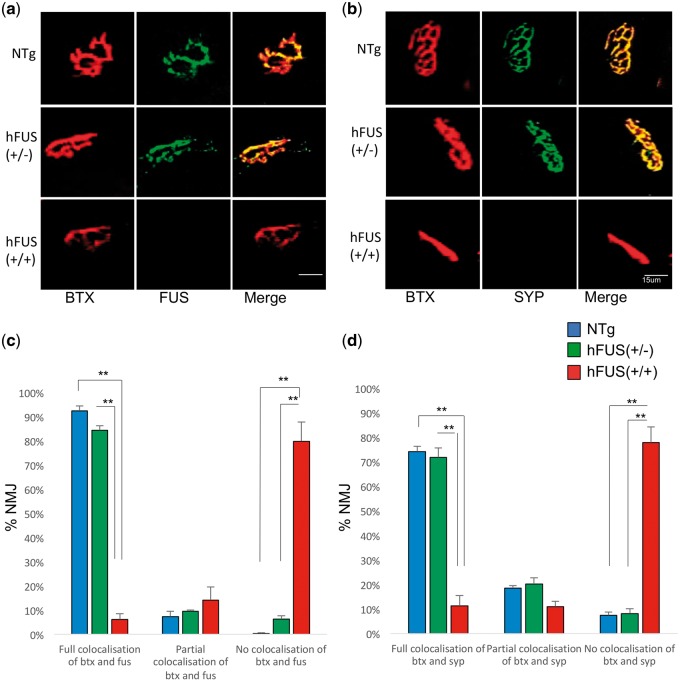
Homozygous hFUS (+/+) transgenic mice show a loss of NMJs by the end stage of disease. (**A, C**) Co-localization of BTX with FUS revealed an abundance of FUS at the NMJs in P56 NTg and hFUS (+/−) transgenic mice whilst homozygous hFUS (+/+) mice, who have severe hind limb paralysis by this age, show a significant decrease in FUS and BTX co-localization (**P < 0.001) (scale bar 15 μm). (**B, D**) Synaptophysin (SYP) staining shows that the NMJ is retained in P56 mice from NTg and hFUS (+/−) mice but that there is a significant loss of SYP and BTX co-localization in hFUS (+/+) animals by P56 (**P < 0.001) (scale bar 15 μm). Significance was determined using a 2-way ANOVA with Holm-Sidak *post hoc* test.

We have previously shown that low level increases in FUS in the hFUS (+/−) mice do not result in any motor impairment or pathological FUS deposition (8), and here we show that these animals also demonstrate no evidence of NMJ loss or pathology by 8 weeks (P56) of age (Fig. 2A and B). In contrast, homozygous hFUS (+/+) mice exhibit a degenerative motor phenotype that necessitates culling by this age (8). Immunohistochemistry in these end stage hFUS (+/+) mice demonstrates major changes in NMJ morphology and peripheral denervation by P56 determined by the significant loss of co-localization between both FUS and BTX [*P* < 0.001 vs NTg and hFUS (+/−) littermates] and the pre-synaptic marker synaptophysin (SYP) and BTX [*P* < 0.001 vs NTg and hFUS (+/−)] littermates) in surviving NMJs (Fig. 2, Supplementary Material, Fig. S1). Staining for neurofilament medium chain (NFM145) confirms denervation of the NMJ in the hFUS (+/+) end stage animals (S2).

### Loss of pre-synaptic protein from neuromuscular junctions at early pre-symptomatic stage P15 without loss of spinal cord motor neurons

**Figure 3.**
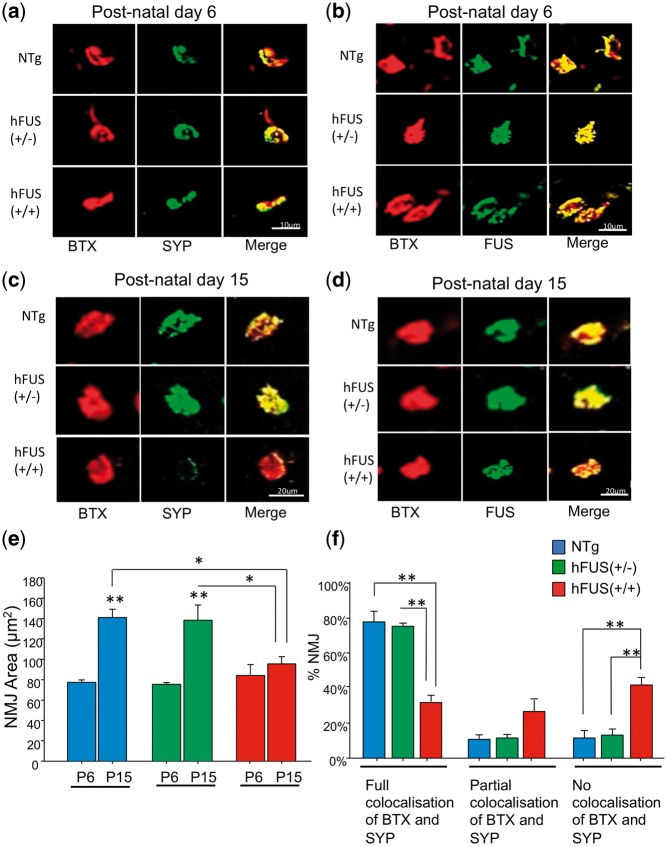
Neuromuscular junction degeneration at post-natal day 15 in hFUS (+/+) mice. (**A,B**) NMJs are fully innervated by SYP with abundant FUS in NTg, hFUS (+/−) and hFUS (+/+) mice at post-natal day 6 (scale bar: 10 μm). (**C,D**) At post-natal day 15, a significant loss of SYP at the NMJs was found in hFUS (+/+) mice, however FUS was still abundant (scale bar: 20 μm). (**E**) There was no significant difference in the average NMJ area between NTg, hFUS (+/−) and hFUS (+/+) mice at post-natal day 6, but a significant difference in the average NMJ area was found in hFUS (+/+) mice at post-natal day 15 [*P = 0.004 vs NTg; *P = 0.005 vs hFUS (+/−)]. Average NMJ area in P15 NTg and hFUS (+/−) mice was significantly increased compared with P6 NTg and hFUS (+/−) mice (**P < 0.001), but not in hFUS (+/+) mice (*P = *0.316). (**F**) In P15 hFUS (+/+) mice, there was a significant decrease in the number of NMJs with complete BTX and SYP overlap (**P < 0.001), and a consummate significant increased number of NMJs with no overlaps of BTX and SYP (*P < 0.001). (*N = *3–4 for all genotypes). Significance was determined using a 2-way ANOVA with a Holm-Sidak *post hoc* test.

In order to investigate early disease events at the NMJ in these animals we performed immunohistochemistry on muscle sections from pre-symptomatic animals. At early pre-symptomatic stage P6, NMJs were fully innervated as indicated by co-localization of BTX and SYP in NTg, hFUS (+/−) and hFUS (+/+) mice (Fig. 3A and Supplementary Material, Fig. S3). In addition, FUS was abundant at the terminals in these animals (Fig. 3B). However, a loss of pre-synaptic protein SYP at the NMJs was observed in hFUS (+/+) mice at a further pre-symptomatic stage P15 (Fig. 3C and Supplementary Material, Fig. S3), with a significant decrease in the number of NMJs with complete overlap between BTX and SYP [*P* < 0.001; hFUS (+/+) vs NTg and hFUS (+/−)] and a significant increase in NMJs with no evidence of BTX and SYP overlap [*P* < 0.001; hFUS (+/+) vs NTg and hFUS (+/−)] (Fig. 3C and F). Although the gross morphology of NMJs in NTg, hFUS (+/−) and hFUS (+/+) mice at P6 was unchanged, by P15 there was a significant difference in the average area of NMJs in hFUS (+/+) mice [*P* = 0.004 vs NTg; *P* = 0.005 vs hFUS (+/−)] (Fig. 3E). Interestingly, FUS was still abundant at the NMJs in hFUS (+/+) mice at P15 (Fig. 3D), suggesting that the loss of SYP in these mice is not simply a marker of the degenerating pre-synaptic terminal, but rather, may reflect a specific aberrant FUS function and a loss of synaptic vesicles due to an over abundance of FUS.

**Figure 4.**
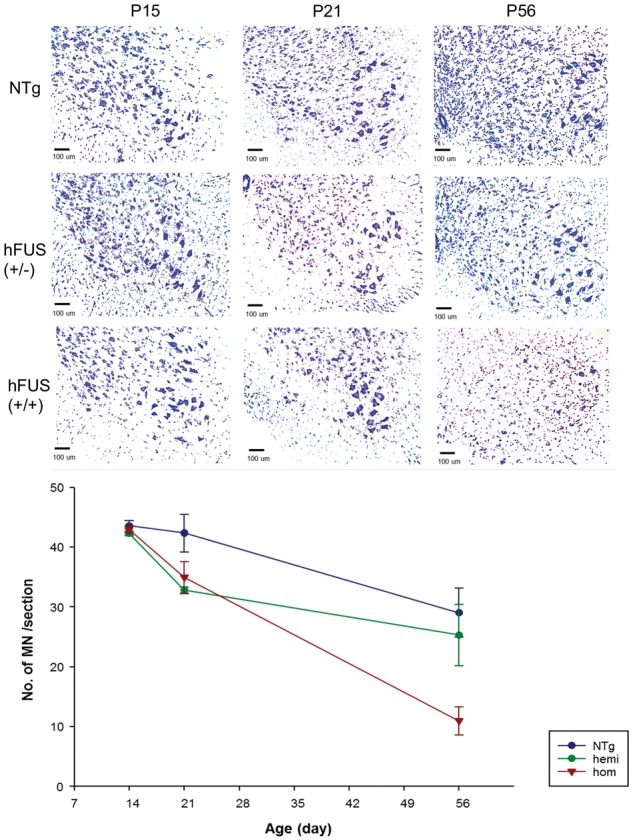
No significant loss of spinal cord motor neurons in hFUS (+/+) mice at post-natal day 15 and day 21. (**A**) Cresyl violet staining of the large diameter α-motor neurons in the ventral horn of the lumbar spinal cord in NTg, hFUS (+/−) and hFUS (+/+) mice at P15, P21 and P56. (**B**) At P15, there was no significant difference in the number of motor neurons between NTg, hFUS (+/−) and hFUS (+/+) mice and hence no neuronal loss in hFUS (+/+) mice despite NMJ degeneration. At P21, there was a non-significant reduction in the number of motor neurons in all three genotypes compared with P15 [NTg *P = *0.78, hFUS (+/−) *P = *0.08, hFUS (+/+) *P = *0.10], which reached significance by P56 [NTg *P = *0.009, hFUS (+/−) *P = *0.002, hFUS (+/+) *P < *0.001]. In hFUS (+/+) mice there was a significant loss of motor neurons compared with NTg and hFUS (+/−) mice at the end-stage P56 [NTg vs hFUS (+/+) *P < *0.001, hFUS (+/−) vs hFUS (+/+) *P = *0.004]. (*N = *3–4 for all genotypes). Significance was determined using a 2-way ANOVA with a Holm-Sidak *post hoc* test.

Despite the observed NMJ degneration in hFUS (+/+) mice at pre-symptomatic stage P15, assessment of large α-motor neurons in the ventral horn of the lumbar spinal cord revealed no significant difference in the number of spinal cord motor neurons at P15 between NTg, hFUS (+/−) and hFUS (+/+) mice [NTg vs hFUS (+/−) *P* = 0.99, NTg vs hFUS (+/+) *P* = 0.99, hFUS (+/−) vs hFUS (+/+) *P* = 0.89] (Fig. 4A and B). Even at a later pre-symptomatic (P21) stage, there is no significant reduction in the number of motor neurons in the hFUS (+/+) mice compared with their hemizygous and NTg littermates [hFUS (+/+) vs hFUS (+/−) *P* = 0.63, hFUS (+/+) vs NTg *P* = 0.19] (Fig. 4A and B). Interestingly, there is a significant decline in motor neuron number from P15 to P56 in all mice [NTg *P* = 0.009, hFUS (+/−) *P* = 0.002, hFUS (+/+) *P* < 0.001], regardless of genotype, suggesting that some form of motor neuronal ‘pruning’ in the spinal cord is a normal developmental event. This pruning appears to be slightly (although not significantly) enhanced in transgenic vs NTg P21 animals [hFUS (+/−) vs NTg *P* = 0.08, hFUS (+/+) vs NTg *P* = 0.10], which may indicate a role for FUS in this process. However, in the hFUS (+/+) mice there was a much larger decrease in motor neuron number by P56 compared with both NTg and hFUS (+/−) animals [NTg vs hFUS (+/+) *P* < 0.001, hFUS (+/−) vs hFUS (+/+) *P* = 0.004] (Fig. 4A and B), indicative of a pathological loss of neurons in these animals that occurs after NMJ degeneration.

### Dramatic ultrastructural changes in the pre-synaptic terminal of pre-symptomatic FUS (+/+) mice

**Figure 5.**
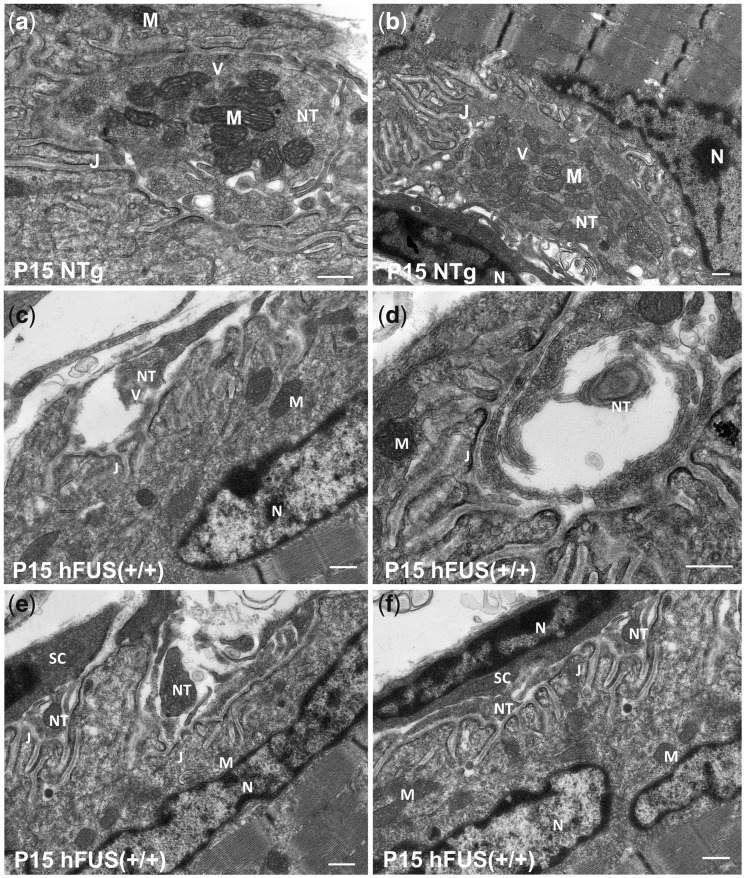
Dramatic ultrastructural changes of the neuromuscular junctions in hFUS (+/+) mice at post-natal day 15. Ultrastructural analyses with TEM revealed dramatic loss of synaptic vesicles and morphological abnormalities at the pre-synaptic nerve terminals in hFUS (+/+) mice, in contrast to NTg mice, in which the axon terminals were full of healthy mitochondria and synaptic vesicles (**A, B**). Pre-synaptic nerve terminals in hFUS (+/+) mice were disrupted and fragmented (**C, E, F**), and sometimes accompanied by the extension of Schwann cell processes into the synaptic cleft, in close contact with the junctional folds (**E, F**). Vacuoles and large empty spaces were also found in some axon terminals (**D**). M, mitochondrion; V, synaptic vesicles; NT, nerve terminal; J, junctional folds; N, nucleus; SC, Schwann cell (scale bar: 500 nm).

**Figure 6.**
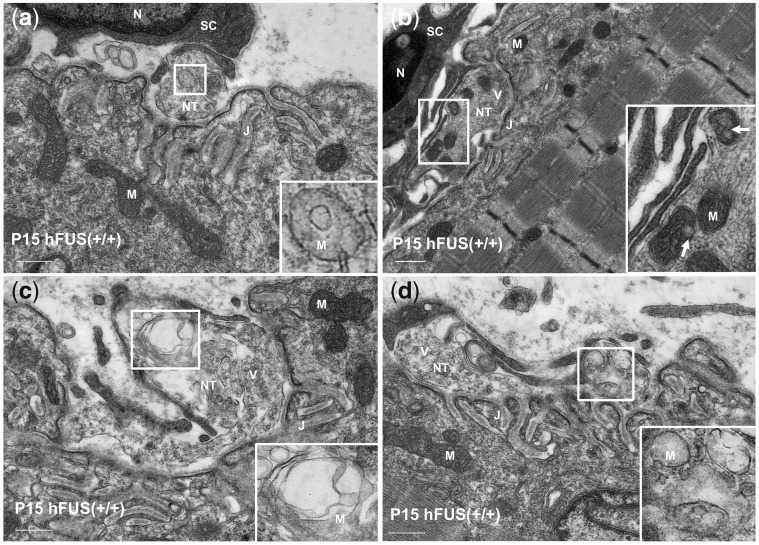
Neuromuscular junctions with striking mitochondrial abnormalities in hFUS (+/+) mice at post-natal day 15. High power TEM images illustrating striking mitochondrial abnormalities at the pre-synaptic terminals at P15, including a reduced mitochondrial density, disorganised cristae (**A**), vacuolation (arrows in **B**), swelling (**C**) and absence of mitochondrial cristae (**D**). M, mitochondrion; V, synaptic vesicles; NT, nerve terminal; J, junctional folds; N, nucleus; SC, Schwann cell (scale bar: 500 nm).

**Figure 7.**
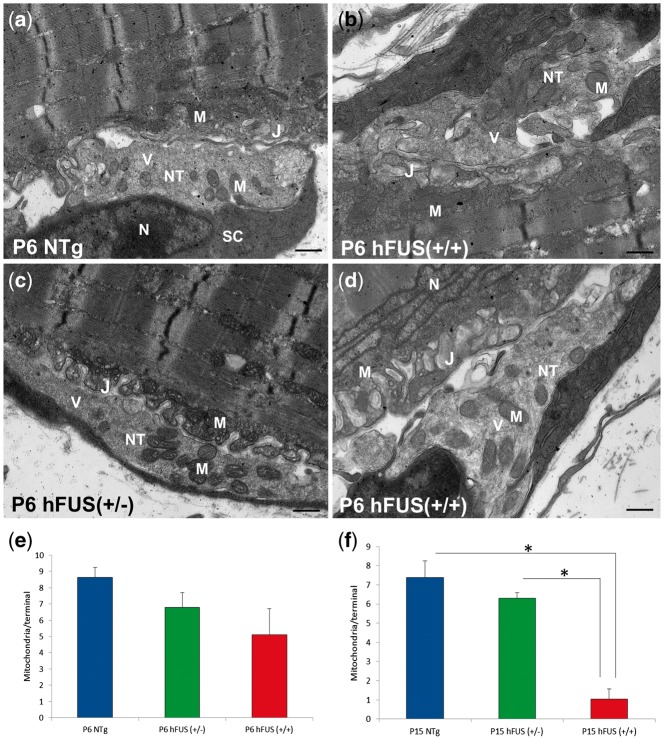
Normal ultrastructural morphology of the majority of neuromuscular junctions in NTg, hFUS (+/−) and hFUS (+/+) mice at post-natal day 6. (**A–D**) TEM images showing the pre-synaptic nerve terminals and post-synaptic muscle endplates of the NMJs. The majority of the pre-synaptic axon terminals contained an abundance of synaptic vesicles and healthy mitochondria in NTg, hFUS (+/−) and hFUS (+/+) mice. M, mitochondrion; V, synaptic vesicles; NT, nerve terminal; J, junctional folds; N, nucleus; SC, Schwann cell (scale bar: 500 nm). (**E,F**) Quantification of the number of mitochondria in the pre-synaptic nerve terminals from P6 and P15 mice shows a significant reduction in the number of mitochondria in P15 hFUS (+/+) animals compare to NTg (*P = 0.003) and hFUS (+/−) animals (*P = 0.008, one-way ANOVA with a Fisher LSD *post hoc* test).

In order to explore pre-symptomatic changes in the ultrastructural morphology at the NMJs we performed TEM in NTg and hFUS (+/+) P15 mice. We observed an abundance of synaptic vesicles and healthy mitochondria at the axon terminals in NTg mice (Fig. 5A and B), however, we found a dramatic loss of synaptic vesicles at nerve terminals of hFUS (+/+) mice which appeared disrupted and fragmented, with multiple vacuoles and empty spaces inside (Fig. 5C and D and Supplementary Material, Fig. S4). On occasion, this was accompanied by the extension of Schwann cell processes into the synaptic cleft to make contact with the post-synaptic junctional folds, indicating complete NMJ denervation (Fig. 5E and F). Quantification of mitochondria at the pre-synaptic nerve terminals using the TEM images revealed a significant decrease in the number in hFUS (+/+) mice at P15 [*P* = 0.003 vs NTg; *P* = 0.008 vs hFUS (+/−); Fig. 7F] while many of those that remained had pronounced abnormalities including disorganised cristae and large vacuoles (Fig. 6A–D). These ultrastructural changes are consistent with our immunohistochemical studies demonstrating the loss of synaptic protein SYP at the NMJs (Fig. 3C) and the significant decrease in the average NMJ area in hFUS (+/+) mice at P15. As expected from the immunohistochemistry data, the hFUS (+/−) mice showed no major structural differences compared with the NTg animals (Supplementary Material, Fig. S5). The mitochondrial abnormalities we observed in our hFUS (+/+) mice are similar to those described in the nerve terminals of aged rats (20), as well as SOD1 and TDP-43 transgenic mice (21–23). It is important to note that despite the profound pre-synaptic mitochondrial abnormalities observed in the hFUS (+/+) mice at P15, mitochondria in the post-synaptic muscle endplate were abundant and of normal appearance (Figs 5 and 6). This is consistent with recent studies demonstrating the vulnerability of axonal mitochondria and showing that distal axon terminals undergo the earliest damage in the course of patients with ALS (24–29). These robust pathological changes in NMJ ultrastructure demonstrate that an increase in cytoplasmic mislocalization of FUS in hFUS (+/+) mice results in major synaptic disruption prior to symptom onset or neuronal loss.

**Figure 8.**
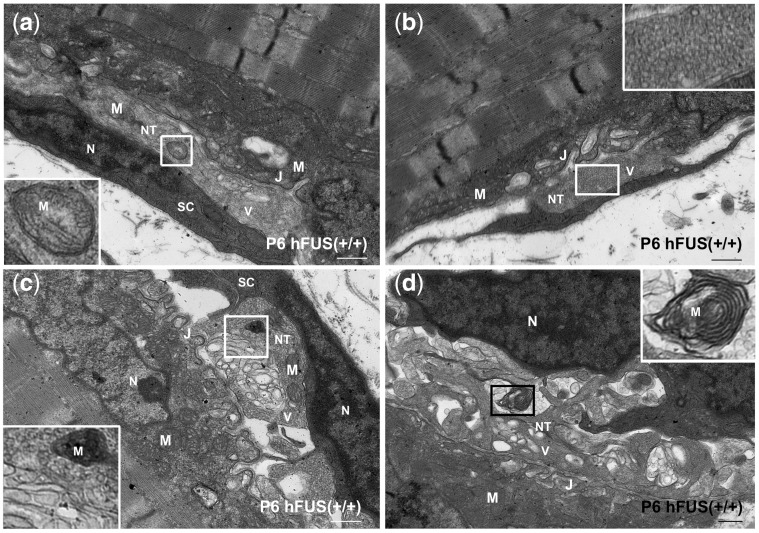
Early mitochondrial abnormalities at the pre-synaptic nerve terminals in a small number of neuromuscular junctions in hFUS (+/+) mice at post-natal day 6. Mitochondrial abnormalities were found at the axon terminals of the hFUS (+/+) mice in a small proportion of NMJs without apparent loss of synaptic vesicles. Mitochondria were lost (**B**) or reduced in density, appeared darker in colour (**C**), with disorganised cristae (**A**) and ‘onion-ring’ membranes (**D**). M, mitochondrion; V, synaptic vesicles; NT, nerve terminal; J, junctional folds; N, nucleus; SC, Schwann cell (scale bar: 500 nm).

To further investigate the earliest aspects of this disruption, we sought to analyse ultrastructural changes at the NMJ in P6 animals. At this age, the majority of the pre-synaptic terminals in NTg, hFUS (+/−) and hFUS (+/+) mice were filled with synaptic vesicles and healthy mitochondria (Fig. 7). This is consistent with our immunohistochemical study showing an abundance of the pre-synaptic vesicle membrane protein SYP at the NMJs in these animals (Fig. 3A). Quantification of these TEM images suggests that P6 hFUS (+/+) mice have around 1/3 less mitochondria than their NTg littermates, although, this reduction did not reach significance (*P* = 0.26, Fig. 7E). Consistent with this finding however, we identified a number of mitochondrial abnormalities at the pre-synaptic terminals in a small number of the NMJs in P6 hFUS (+/+) mice that were never observed in NTg or hFUS (+/−) mice (Fig. 8). Some of the mitochondria present showed vacuolation, an abnormal arrangement of cristae and ‘onion-ring’ membranous structures (Fig. 8A, C, D respectively). These are recognised features of mitochondrial degeneration and suggest that mitochondrial dysfunction may be one of the prime drivers of FUS-linked disease.

## Discussion

Many strands of evidence suggest that synaptic dysfunction and disruption at the NMJ is one of the earliest events in ALS and leads to muscle atrophy that is the hallmark of this disease (15,16,18). Our understanding of the mechanisms driving NMJ dysfunction largely come from studies on mutant SOD1 in transgenic mice and TDP-43 and FUS toxicity in *Drosophila* and zebrafish (16,19,30–35). Here, we report that in mice, endogenous FUS is abundant at the NMJ and that overexpression of human wild-type FUS causes a progressive loss of NMJs. This was evident at post-natal day 15, prior to the earliest motor phenotype of tremor and altered gait, which appears around P28 (8) and before motor neuron loss in the spinal cord was detected. NMJ loss was accompanied by striking ultrastructural changes at the pre-synaptic terminals including the loss of synaptic vesicles and a reduced number of mitochondria, with many of those remaining having major structural abnormalities. More subtle features of mitochondrial vacuolation and disordered cristae and membranes were already present at post-natal day 6. These findings indicate that FUS plays an important role in synaptic function at the NMJ, and that FUS overexpression causes mitochondrial disruption, NMJ loss and a ‘dying-back’ axonopathy, which eventually leads to motor neuron degeneration. This sequence of events is similar to those observed in mutant SOD1 and TDP-43 mediated NMJ degeneration (16,19,30,32) and indeed central synaptic degeneration observed in transgenic models of Alzheimer’s and Parkinson’s (36–38). Interestingly FUS-related degeneration bears a striking similarity to that seen in Spinal Muscular Atrophy (SMA). FUS and SMN, the loss of which causes SMA, are both RNA-binding proteins involved in similar cell processes including splicing. They have been shown to interact with each other and SMN has been found in cytoplasmic mutant-FUS aggregates (39,40). Indeed studies using the same hFUS overexpressing mouse line reported here have shown that there are changes in RNA splicing and the spliceosome that are similar to those seen in SMN mouse models (41). Further mouse models of both diseases have now been shown to have degeneration of the NMJ as an early disease event (42,43). Given the many links between FUS and SMN, it is conceivable that the two diseases share a common pathomechanism.

Our observation that FUS is abundant at the NMJ emphasises the important role that FUS plays outside the nucleus, particularly in the transport of mRNA into axons and dendrites and the regulation of local translation of synaptic proteins (11,44). FUS has previously been observed in close proximity to synaptic vesicles within the axon terminal of rat hippocampal neurons using super-resolution microscopy (45). Furthermore, dysfunction and disruption of cortical synaptic connections have previously been reported following FUS knock-down and overexpression (14,44). Here, however, we show that increasing FUS concentration by a factor of 1.7 above endogenous (8) is sufficient to cause a devastating effect in the peripheral nervous system at the NMJ.

In order to define the sequence of events that lead to motor neuron degeneration, we performed immunohistochemical studies on NMJs in pre-symptomatic animals in the gastrocnemius muscle and correlated this with motor neuron counts in the lumbar spinal cord. A significant decrease in the average surface area of NMJs was accompanied by a significant loss of the pre-synaptic protein synaptophysin in hFUS (+/+) mice at P15 that was not detectable at P6. Surprisingly, FUS protein was still abundant at the pre-synaptic terminal in NMJs at P15, suggesting that the loss of synaptophysin is not due to complete nerve terminal loss, but rather that FUS overexpression may impair synaptic protein translation. The association of FUS with cytoplasmic stress granules suggests it may act as an active repressor of translation (46,47), thus the overexpression of FUS may directly repress translation of synaptophysin and other pre-synaptic proteins in homozygous mice or aberrantly process other mRNAs at the synaptic terminals, causing synaptic defects by a toxic gain-of-function. Despite the observed NMJ degeneration in hFUS (+/+) mice at P15, there was no significant motor neuron loss in the lumbar spinal cord compared with NTg and hFUS (+/−) mice at this stage. Interestingly, we observed a reduction in motor neuron number in all mice, regardless of genotype, from 15 to 56 days of age, suggesting that there are post-natal developmental refinements of the rodent motor system, whereby extraneous cells are removed. Certainly, studies have shown there are critical post-natal periods for the development of normal motor function in the rodent (48). The subtle differences we observed in the profile of motor neuron reduction over this time between the transgenic and NTg animals, with both the hFUS (+/−) and hFUS (+/+) animals showing a greater reduction by P21, may suggest that FUS has a role to play in this developmental process, and indeed, this early change may be indicative of an increased susceptibility to disease in adulthood.

Although all animals displayed a reduction in motor neurons between 15 and 56 days of age, this reduction was significantly enhanced in a pathological fashion in the symptomatic hFUS (+/+) mice, compared with the non-symptomatic NTg and hFUS (+/−) animals, confirming that motor neuron degeneration in these WT-overexpressing mice begins in the periphery well before motor neuron loss is observed in the spinal cord. This finding supports our hypothesis that the disease process in these animals is a ‘dying-back’ axonopathy mechanism similar to that reported in other ALS gene animal models and some human data (15,16,19,32).

The vast majority of studies describing a ‘dying-back’ axonopathy mechanism in ALS are based on mutant SOD1 models (15,16,19) and relatively little is known about disease mechanisms for FUS-linked ALS and FTLD. Recent studies in *Drosophila* and zebrafish indicate that loss and gain of toxic FUS function may cause early defects in axonal transport, pre-synaptic structure and transmission at the NMJ preceding motor neuron degeneration (31,33–35). Motor neuron degeneration preceded by structural and functional changes at the NMJ has recently been reported in FUS transgenic mice (13). Interestingly, a motor neuron degeneration phenotype was only observed in mice expressing mutant FUS and not in the hemizygous or homozygous mice expressing wild-type FUS, which indicated a mutant-dependent toxicity. However, wild-type FUS accumulation has been found in both FTLD and ALS patients, and studies in rodents also demonstrated that increased expression of wild-type FUS is pathogenic and causes neurodegeneration (8,49). Further, there are reported cases of FUS mutations in the 3’UTR of the gene that result in an increased protein expression that mislocalises to the cytoplasm (50). This is further supported by reports of wild-type FUS toxicity in other species (33,51,52). Furthermore, in contrast to our mice, the mutant FUS expressing mice exhibited a slowly progressive phenotype, with both pre- and post-synaptic deficits being reported (13). These differences may be due to different promoter systems, expression levels and background strain variations in comparison with our mice.

The earliest ultrastructural changes at the NMJ in hFUS (+/+) mice were to mitochondria at the pre-synaptic nerve terminals. These were subtle at P6, involving vacuolation and membrane abnormalities, but were much more pronounced by P15 with a significant loss of morphologically normal mitochondria and synaptic vesicles. Interestingly, there is a trend towards a decrease in the number of mitochondria in hFUS (+/−) animals at both P6 and P15 despite these animals being asymptomatic out to 2 years of age. However, it should be noted that quantification of the TEM images is only semi-quantitative and further investigations will be needed to determine how significant this is for disease. Mitochondrial dysfunction is increasingly recognised as a converging point for many pathological pathways in ALS (22,28,53–55), and has previously been described in ALS-FUS patients and other disease models (54,56,57). A number of mitochondrial proteins have been identified as FUS interacting partners (53) and FUS regulates the expression of several genes involved in mitochondrial biogenesis, homeostasis and function (58,59). Mitochondrial defects following FUS overexpression may therefore be due to dysregulation of mitochondrial, synaptic or cytoskeletal genes, but may also be due to direct toxicity of the FUS protein to mitochondria. Identifying whether the overexpression of FUS causes the mitochondrial dysfunction and loss of synaptic vesicles directly or whether these are simply symptoms of a sick motor neuron is a crucial next step in determining early disease events. Whatever the mechanisms our results support the hypothesis that mitochondrial dysfunction and early degeneration of the NMJ is a convergent event in the pathogenesis of ALS (26,28,29).

## Materials and Methods

### Ethics statement

Experiments were carried out under the UK Animals (Scientific Procedures) Act 1986, and were approved by the Kings College, London ethics review panel.

### Transgenic mice

Transgenic animals were generated and genotyped as described in Mitchell *et al.*, 2013 (8), and were maintained on a C57Bl/6J background for all experiments. Mice were housed in a 12 h: 12 h light: dark cycle, with ad libitum access to food and water. Homozygous animals were provided with wet mash from symptom onset to help prolong survival. 

### Histology and immunohistochemistry

P21 and P56 homozygous FUS mice and their age-matched hemizygous and non-transgenic littermates were anaesthetised and perfused transcardially with phosphate buffer followed by 4% paraformaldehyde (PFA) in PBS. Brain, spinal cord and gastrocnemius muscles were dissected out and postfixed in 4% PFA in 15% sucrose for 5 h, followed by cryoprotection in 30% sucrose for 24 h. Brain and spinal cord were cut on a cryostat into 30 μm thick sections and muscles were cut into 40 μm thick sections. For post-natal day 6 and day 15 mice, brain, spinal cord and gastrocnemius muscles were dissected and immersion-fixed in 4% PFA overnight, then 4% PFA in 15% sucrose for 5 h, cryoprotected in 30% sucrose for 24 h.

For Immunohistochemistry and the detection of NMJs, 40 μm thick sections of gastrocnemius muscle were incubated in the following antibodies: Alexa Fluor 555 α**-**bungarotoxin (1: 500, Life Technologies), rabbit anti-Synaptophysin 1 (1: 500, Synaptic Systems), rabbit anti-TDP-43 (1: 1000, Proteintech), rabbit anti-FUS (1: 200, Sigma) and rabbit anti-Neurofilament M (145 kDa) (1: 150, Millipore). Tissues from three animals were taken for each genotype [non-transgenic, hemizygous FUS (+/−) and homozygous FUS (+/+)] at each age group. Muscle sections were then washed and further incubated in donkey anti-rabbit IgG DyLight 488 secondary antibody (1: 500, Thermo Scientific) and imaged with a Leica confocal microscope and LAS-AF software. To assess the area of NMJs, total area stained by α**-**bungarotoxin was measured in 30–40 NMJs per animal using ImageJ. To assess the co-localization of pre- and post-synaptic membrane proteins at the NMJ, 70–100 NMJs were analysed per animal per genotype for all ages and full or partial co-localization of α**-**bungarotoxin and synaptophysin or FUS and α**-**bungarotoxin were assessed by eye. 

#### Super-resolution microscopy

Tissue was processed for normal immunohistochemistry and then imaged and super-resolved images collected using a Visitech-iSIM module coupled to a Nikon Ti-E microscope using a Nikon 100x 1.49NA TIRF oil immersion lens. Green fluorescence was excited with a 488 nm laser and emission filtered through a 525/25 filter. Red Fluorescence was excited with a 561 nm laser and emission filtered through a 630/30 filter. Multiple images at focal planes were collected spaced apart by 0.05 µm. Data were deconvolved using a Richardson-Lucy algorithm specific to the iSIM mode of imaging to increase contrast and resolution using the supplied Nikon deconvolution software.

#### Motor neuron count

Motor neuron counts in post-natal day 15, 21 and 56 mice were performed in 3 or 4 animals per genotype. 4% PFA fixed lumbar spinal cords were sectioned serially into 30 μm thick sections and every sixth section was analysed. Sections were mounted onto slides and left to air dry. To de-fat the sections, they were incubated in 1: 1 ethanol/chloroform overnight. Sections were stained in 0.1% cresyl violet at 37 °C for 10 min, dehydrated, mounted with DPX and sealed with coverslips. MetaMorph 7.7, Molecular Devices, Wokingham, UK was used to count and compare the number of spinal cord motor neurons. Large neurons with diameter greater than 30 μm in the anterior horn of the lumbar spinal cord were counted using the integrated morphometry analysis package in 30 sections per animal.

#### Conventional electron microscopy

Gastrocnemius muscles were dissected from post-natal day 6 and day 15 mice, immersion-fixed in 4% PFA and 0.1% gluteradehyde in 0.1 m phosphate buffer (pH 7.3) overnight at 4 °C. The muscles were sliced into 150 μm thick sections with a vibratome and stained with biotinylated α-BTX (1: 250) overnight at 4 °C. Sections were washed in 0.1 m phosphate buffer then incubated in streptavidine-HRP for 10 min at room temperature, followed by DAB staining for 10 min. Regions of the samples with clusters of NMJs were identified and micro-dissected into smaller fragments under light microscope and processed for TEM.

Samples were washed in 0.1 m phosphate buffer and further post-fixed in 1% osmium tetroxide in phosphate buffer at 4 °C for 1.5 h, then washed and dehydrated through a graded series of ethanol, and then equilibrated with propylene oxide followed by infiltration with epoxy resin (TAAB Laboratories Equipment, Reading, UK). Samples were cut into smaller pieces prior to embedding in epoxy resin and the embedded samples were left in an oven to polymerize at 70 °C for 24 h. Ultrathin sections around 70–90 nm were cut using the ultramicrotome (Reichert-Jung Ultracut E, Leica) and mounted on 150 mesh copper grids. Grids were then stained with uranyl acetate and lead citrate to enhance the contrast. The FEI Tecnai 12 transmission microscope was used for sample imaging and images were captured using an AMT 16000M camera.

#### Mitochondria count

The number of mitochondria in the pre-synaptic nerve terminal was counted with the TEM images of the NMJs. On average 10 NMJs were assessed for mitochondria count in each animal and 2–3 animals per genotype at post-natal day 6 and 15 were assessed.

### Statistical analyses

The data are presented as mean ± standard error of the mean (SEM). A *P* value of <0.001 is considered to be highly significant, and *P* value of <0.05 significant. Statistical significance was determined using either one-way or two-way analysis of variance (ANOVA), followed by either a *post hoc* Holm-Sidak test (Fig. 1), a *post hoc* Tukey test (Figs 2 and 3) or a Fisher LSD *post hoc* test (Fig. 6).

## Supplementary Material

Supplementary Material is available at *HMG* online.

*Conflict of Interest statement*. None declared. 

## Funding 

Motor Neuron Disease Association UK; The Wellcome Trust; NIHR Biomedical Research Centre for Mental Health; The South London and Maudsley NHS Foundation Trust; Medical Research Council UK; The Psychiatry Research Trust of the Institute of Psychiatry Psychology and Neuroscience; Lily Safra Hope Foundation (to E.S.). Funding to pay the Open Access publication charges for this article was provided in equal measure by the RCUK and COAF block grants held by King s College London and part of the open access agreement by MRC (MR/L021803/1) and MRC Wellcome trust (089701/Z/09/Z).

## Supplementary Material

Supplementary Figures
