# Supplementary material for: Mitochondrial abnormalities and disruption of the neuromuscular junction precede the clinical phenotype and motor neuron loss in hFUSWT transgenic mice
Source: Hum Mol Genet. 2017 Nov 28;27(3):463–74. doi: 10.1093/hmg/ddx415 (PMC5886082; doi:10.1093/hmg/ddx415)
Supplement: Supplementary Figures [file supplementary_data_ddx415.pdf]

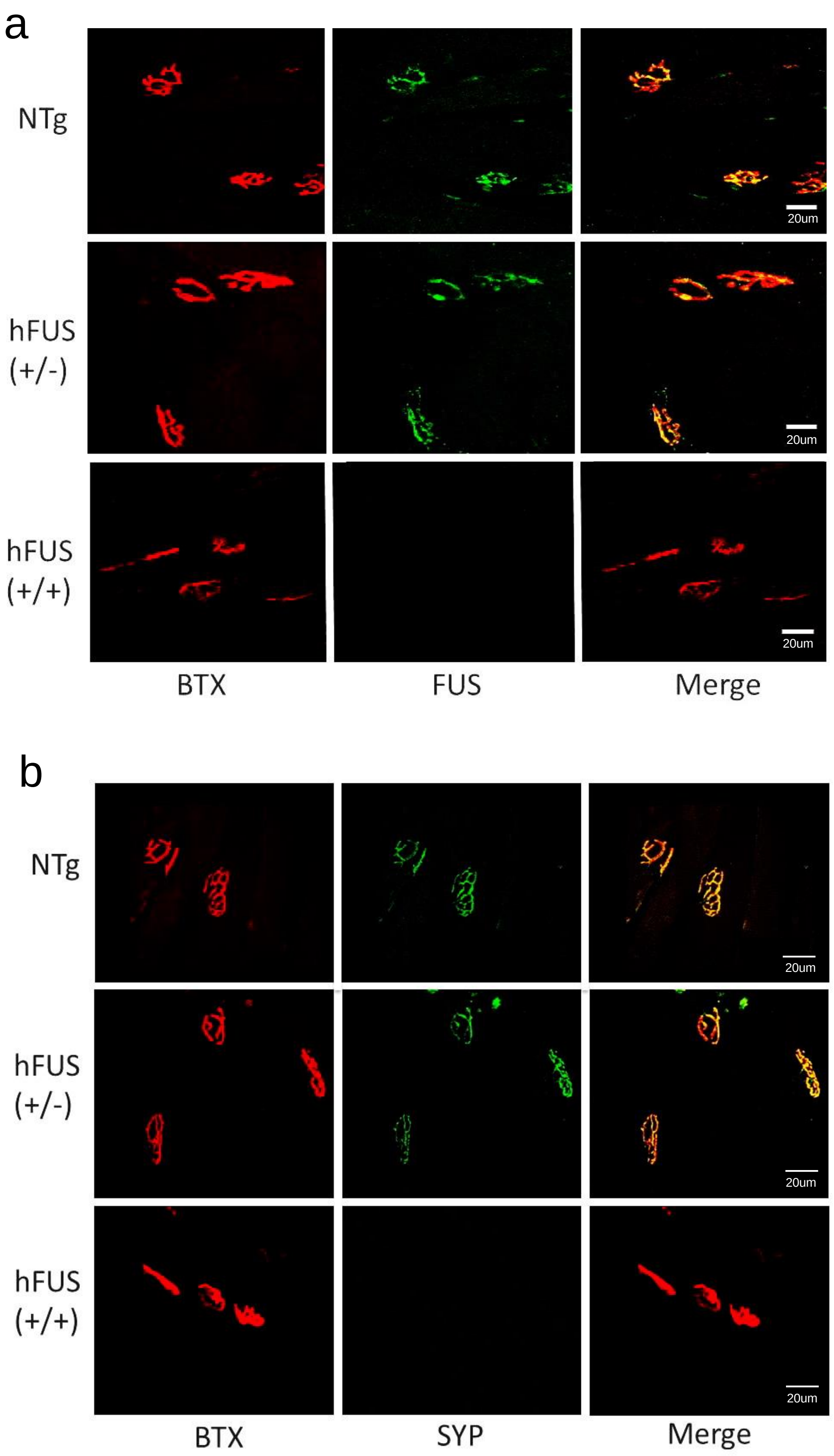

Figure S1: Co-localisation of BTX and FUS revealed an abundance of FUS at the NMJs in P56 NTg and hFUS(+/-) mice (a). NMJs in these mice are fully innervated by synaptophysin (SYP) (b). However in P56 hFUS(+/+) mice there is a complete loss of FUS (a) and SYP (b) at their BTX positive NMJs.

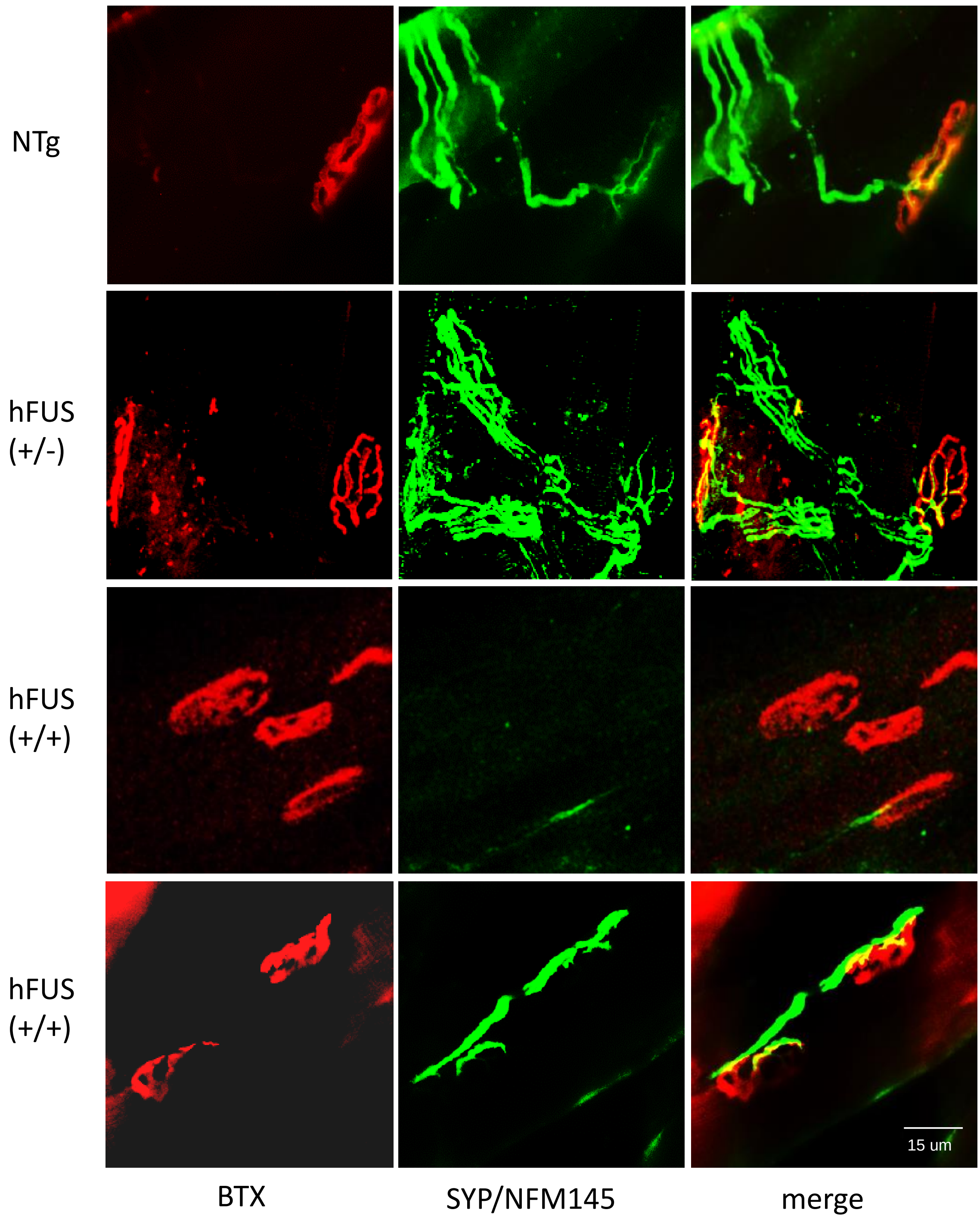

Figure S2: Co-staining of pre-synaptic SYP and neurofilament NFM145 (green) at P56 showing the motor axons innervate the NMJs in both NTg and hFUS(+/-) mice, whereas in hFUS(+/+) animal there is a complete loss of SYP and either a loss of neurofilament or accumulated neurofilament that does not fully innervate the NMJs, indicating NMJ denervation in the end stage hFUS(+/+) animal.

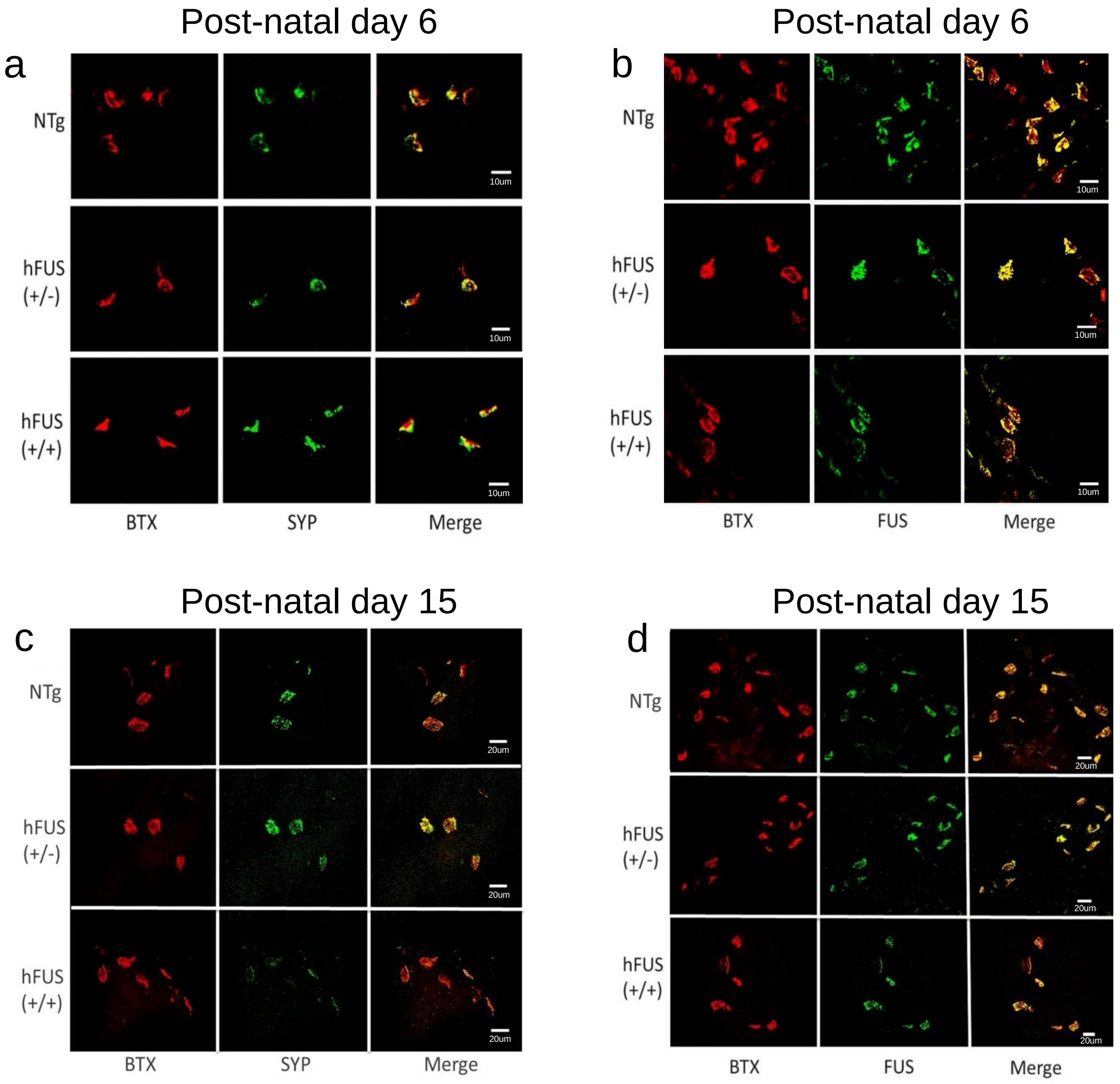

Figure S3: NMJs are fully innervated by SYP with abundant FUS in NTg, hFUS(+/-) and hFUS(+/+) mice at post-natal day 6 (a-b). At post-natal day 15, a loss of SYP staining was found in hFUS(+/+) mice (c), however FUS was still abundant at the NMJs (d).

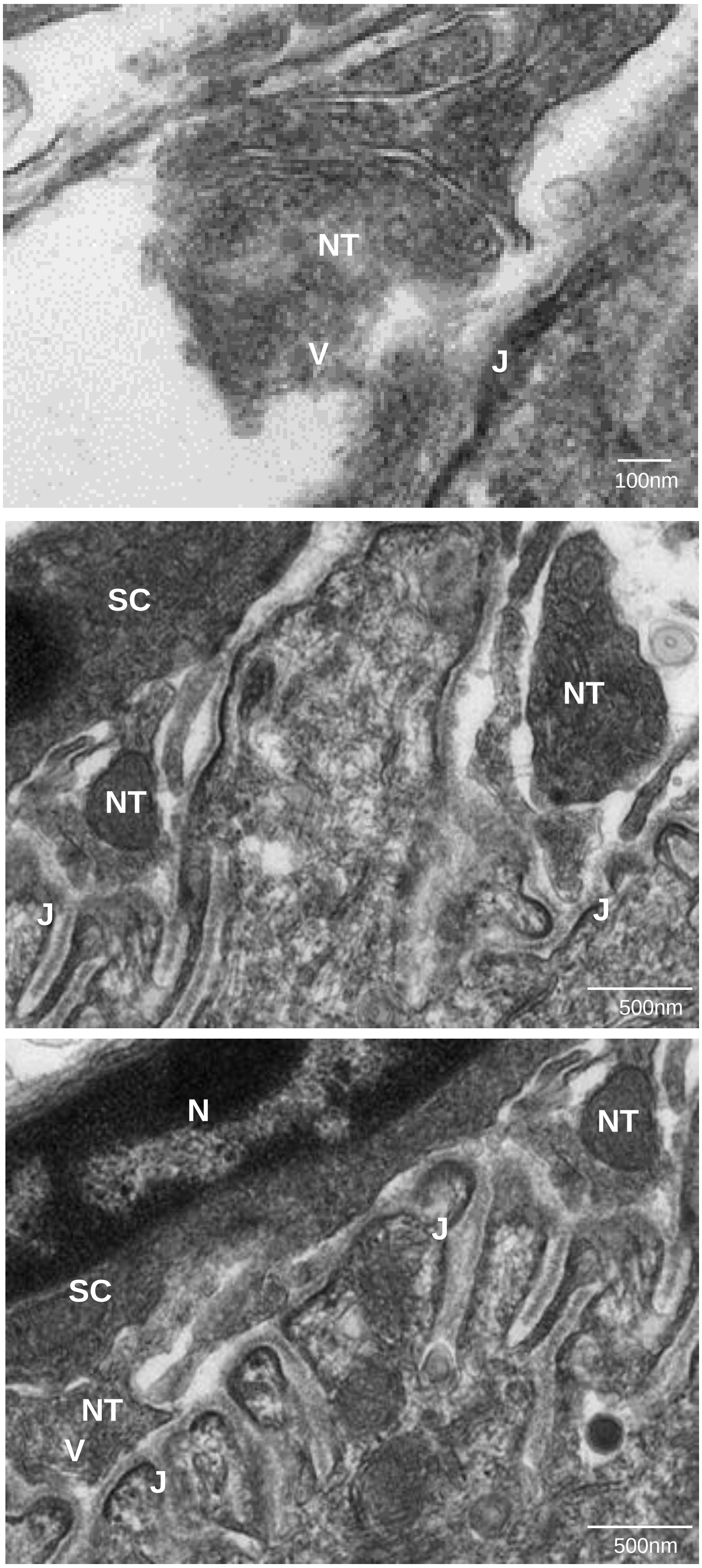

Figure S4: High power TEM images of the fragmented presynaptic nerve terminals in hFUS(+/+) mice at post-natal day 15. V, synaptic vesicles; NT, nerve terminal; J, junctional folds; N, nucleus; SC, Schwann cell

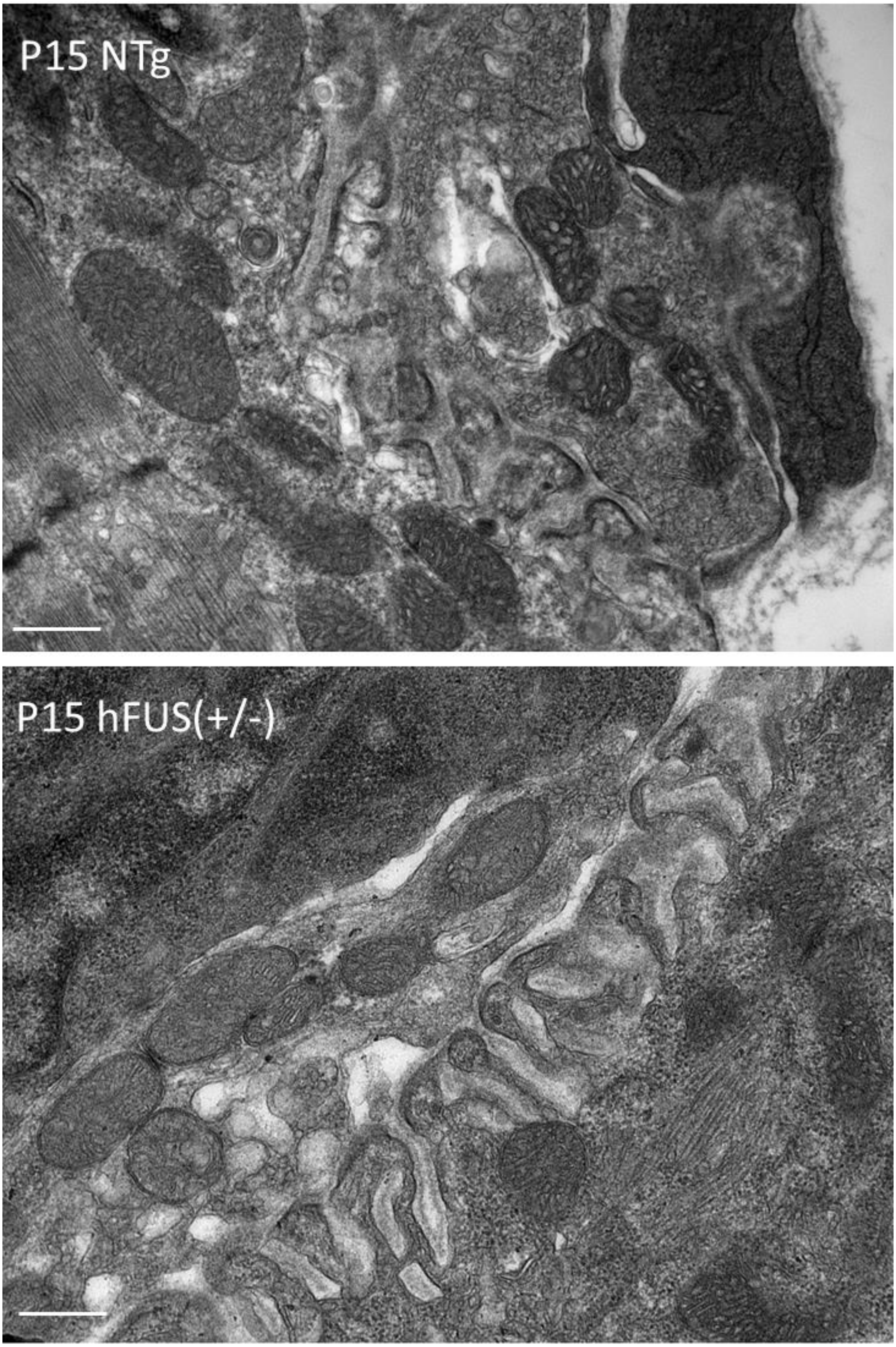

Figure S5: TEM image of a NMJ from the hFUS(+/-) animal showing an abundance of normal mitochondria and synaptic vesicles with distinct junctional folds at post-natal day 15. This looks similar to the NMJ from a NTg animal as seen above. (scale bar: 500nm)
